# Subtypes of relapsing-remitting multiple sclerosis identified by network analysis

**DOI:** 10.3389/fdgth.2022.1063264

**Published:** 2023-01-11

**Authors:** Quentin Howlett-Prieto, Chelsea Oommen, Michael D. Carrithers, Donald C. Wunsch, Daniel B. Hier

**Affiliations:** ^1^ Department of Neurology and Rehabilitation, University of Illinois at Chicago, Chicago, IL, United States; ^2^ Department of Electrical and Computer Engineering, Missouri University of Science and Technology, Rolla, MO, United States

**Keywords:** multiple sclerosis, phenotype, network analysis, communities, modularity, subtype, feature reduction, subsumption

## Abstract

We used network analysis to identify subtypes of relapsing-remitting multiple sclerosis subjects based on their cumulative signs and symptoms. The electronic medical records of 113 subjects with relapsing-remitting multiple sclerosis were reviewed, signs and symptoms were mapped to classes in a neuro-ontology, and classes were collapsed into sixteen superclasses by subsumption. After normalization and vectorization of the data, bipartite (subject-feature) and unipartite (subject-subject) network graphs were created using NetworkX and visualized in Gephi. Degree and weighted degree were calculated for each node. Graphs were partitioned into communities using the modularity score. Feature maps visualized differences in features by community. Network analysis of the unipartite graph yielded a higher modularity score (0.49) than the bipartite graph (0.25). The bipartite network was partitioned into five communities which were named fatigue, behavioral, hypertonia/weakness, abnormal gait/sphincter, and sensory, based on feature characteristics. The unipartite network was partitioned into five communities which were named fatigue, pain, cognitive, sensory, and gait/weakness/hypertonia based on features. Although we did not identify pure subtypes (e.g., pure motor, pure sensory, etc.) in this cohort of multiple sclerosis subjects, we demonstrated that network analysis could partition these subjects into different subtype communities. Larger datasets and additional partitioning algorithms are needed to confirm these findings and elucidate their significance. This study contributes to the literature investigating subtypes of multiple sclerosis by combining feature reduction by subsumption with network analysis.

## Introduction

Multiple sclerosis (MS) is one of several immune-mediated demyelinating diseases of the central nervous system that includes transverse myelitis, optic neuritis, neuromyelitis optical, acute disseminated encephalomyelitis, and acute hemorrhagic leukoencephalopathy (1). MS has traditionally been divided into four clinical course phenotypes that include relapsing-remitting multiple sclerosis (RRMS), primary progressive multiple sclerosis (PPMS), secondary progressive multiple sclerosis (SPMS), and relapsing progressive multiple sclerosis (RPMS) (2). In 2013, the criteria for MS phenotypes were revised to remove RPMS (3–6). More recently, MS has been additionally classified by activity (active or inactive) or phase (relapsing or progressive) (4, 5, 7, 8). Another way of subtyping neurologic diseases is by deep phenotyping where signs and symptoms are recorded in detail and are mapped to a restricted terminology (9–13). Patients can then be subtyped according to patterns of signs and symptoms.

MS may have a variable onset with the diverse symptoms of optic neuritis, facial pain, hemifacial spasm, Lhermitte’s sign, transverse myelitis, limb weakness, limb numbness, urinary retention, dysmetria, intention tremor, incoordination, dysarthria, hearing loss, color blindness, gait disturbance, and diplopia (14). MS variably involves the optic nerve (painful loss of vision), the spinal cord (sphincter dysfunction, monoparesis, hemiparesis, hypoesthesia, paresthesia), the brainstem and cerebellum (diplopia, oscillopsia, vertigo, ataxia, tremor, facial weakness), or the cerebral hemispheres (hemiparesis, hemihypoesthesia) (8). Subtypes of multiple sclerosis based on clinical presentation (signs and symptoms) are recognized (15–18) including tremor (19), ataxia (20), visual disturbances (21, 22), sensory symptoms (numbness and paresthesias) (23–26), pyramidal tract findings (weakness, hyperreflexia, spasticity, and hypertonia) (27–29), or spinal cord findings (paraparesis, sphincter dysfunction, and sensory levels) (30, 31). Other MS subjects show cognitive impairment (32, 33), dysarthria (34), dysautonomia (35), depression (36), imbalance (37), paroxysmal symtoms (38), or fatigue (39, 40).

The Kurtzke Functional System Score (FSS) (41) is useful in rating sensory, visual, sphincter, mental, pyramidal, cerebellar, and brainstem dysfunction in MS. However, there is a limited ability to categorize MS subjects based on their predominant clinical presentation. A network analysis of subjects with MS based on their signs and symptoms could assist in identifying clinically significant subtypes of MS.

This paper is organized as follows. We first review prior work on finding subtypes of multiple sclerosis based on signs and symptoms. We then describe our proposed approach to finding subtypes of multiple sclerosis based on deep phenotyping, subsumption of phenotype classes into superclasses, and network analysis. In the Methods section, we describe how deep phenotyping was performed, how the features were collapsed into superclasses, and how the networks were created and partitioned. In the Results section, we report the partitioning of the networks into five communities of MS subjects. In the Discussion section, we discuss the identified communities as possible clinical subtypes of MS. Finally, we discuss the limitations of network analysis as a method of finding MS subtypes.

### Prior work

Although network analysis has not been used to identify clinical subtypes of MS, other work is relevant to this undertaking (Table 1). Depression and anxiety have been reported in MS in about 27% of patients, but no specific phenotype has been described (42, 43, 62). Zhang et al. (63) examined 13 common symptoms of MS in 1985 MS subjects and found that depression, pain, and walking difficulties were the strongest predictors of impaired quality of life. Cognitive impairment is frequent in MS, possibly affecting 40–80% of subjects (33, 45–50). Pure cognitive subtypes (cognitive impairment without other major neurological signs) have been described in a small minority of MS patients (47, 50). In their review of functional connectivity based on functional MRI, Tahedl et al. (64) suggested that cognitive impairment in MS was associated with disruptions of the default-mode network of the brain, whereas sensory-motor deficits were associated with disruptions of the sensory-motor networks of the brain. Although fatigue is frequent in MS, specific subtypes have not been described (51).

**Table 1:** Summarization of prior work relevant to subtyping multiple sclerosis by phenotypic feature.

Author	Domain	N	Cite	Findings	Limitations
Donnchadha	Behavioral	33	(42)	27% had anxiety	Small N, no correlations with other features
Koch	Behavior	1,376	(43)	27% depressed, stable over time	No correlation with EDSS
Ganesvaran	Brainstem or cerebellum	20	(44)	80% of 20 MS patients with psoriasis 80% had brainstem or cerebellar lesions	Small N
Naismith	Cerebellar and gait	166	(17)	Compared to Whites, Blacks had more gait and cerebellar deficits	Small N
Rocca	Cognitive	227	(45)	Loss of connectivity predicts memory and attention deficits	Correlation with cognitive impairment
Hancock	Cognitive	1,281	(46)	Cognitive domain impairment: 48% intact, 22% 1-domain, 24% 2-domain, 15% multi-domain cognitive deficits	Only examined cognitive impairment
De Meo	Cognitive	1,212	(33)	5 cognitive subtypes identified Only 19.5% completely normal	Only examined cognitive impairment
Staff	Cognitive	23	(47)	Mayo Clinic reported 23 MS patients with isolated cognitive impairment	Population not reported
Leavitt	Cognitive	128	(48)	43.7% cognitively impaired Memory, Processing speed, or both	Convenience sample
Cabeça	Cognitive	35	(49)	Discriminant analysis found reaction time best discriminator	Study did not identify cognitive subtypes
Zurawski	Cognitive	2,302	(50)	2.6% had pure cognitive phenotype	Only examined cognition
Beckerman	Fatigue	264	(51)	88 with low and 174 with high fatigue physical from mental fatigue correlated	Did not correlate fatigue with other features
Bove	FSS^a^	1,028	(52)	Median and range FSS provided	No classification by subtype
Kalincik	FSS^a^	14,969	(53)	Increased disability with relapse on on pyramidal, cerebellar, sphincter FSS	Not classified by phenotype
Stewart	FSS^a^	19,504	(54)	Pyramidal, cerebellar, sphincter add to disability with relapse	Not classified by phenotype
Scott	FSS^a^	1,173	(55)	On followup, most worsening on pyramidal, sensory, cerebellar, and sphincter FSS scales	Not classified by phenotype
Revil	Pain	112	(56)	40 pain free with normal sensation 44 central pain with hyposensitivity	Only examined pain
Tsantes	Relapse phenotype	199	(57)	47% of relapses recurred at initial optic, spinal, brainstem-cerebellum sites	Small N
Mowry	Relapse phenotype	195	(58)	Relapse more likely to recur at optic nerve spinal cord, or brainstem-cerebellum	Small N
Deen	Relapse phenotype	190	(59)	Relapse recurred at optic nerve brainstem-cerebellum, spinal cord	Small N
Nociti	Spinal cord	563	(30)	13/563 had spinal MS (2.3%)	Retrospective study
Sanders	Sensory	127	(26)	84% had paresthesias	Small N
Cree	Spinal cord	1490	(60)	Compared to White, Black MS subjects more corticospinal and transverse myelitis	
Ayache	Tremor	32	(19)	56% with tremor	Not population-based
Gerbis	Vision	550	(61)	5 of 550 patients had severe optic neuritis that never recovered	Only examined optic neuritis

Author is the first author, N is the number of subjects in the study, cite is the reference number. Studies are sorted by domain.

^a^ Functional system score.

Although uncommon, spinal MS (leg weakness, sphincter dysfunction, sensory levels, spasticity, and hyperreflexia), as well as opticospinal MS (combining spinal MS with optic nerve involvement), are recognized forms of MS (65–67). Opticospinal MS must be differentiated from neuromyelitis optica, a similar but etiologically different disease from MS. Cree et al. (60) have suggested that spinal MS and opticospinal MS may be more common in Blacks than Whites. Nociti et al. (30) reported spinal MS in 2.3% of their cohort of subjects.

Cerebellar and brainstem phenotypes of MS have been reported (44) with prominent ataxia and cranial nerve deficits. Naismith et al. (17) compared 79 Black subjects with MS to 80 White subjects (17) and found more tremor, ataxia, and need for assistive walking devices in the Black MS subjects. They speculated that the optico-spinal, cognitive, and ataxic-spastic phenotypes are more common in Black than White subjects. In a small study, Ayache et al. (19) found tremor in 56% of their cohort of MS subjects but did not identify a specific phenotype.

Sensory symptoms are common in MS, including pain, hypesthesias hyperesthesias, band-like sensations, and paresthesias (26, 56); however, no specific sensory phenotype has been described. Optic neuritis is common in MS but generally recovers fully or partially. Gerbis et al. (61) describe 5 subjects from a cohort of 550 MS who had severe unilateral optic neuritis without recovery, and suggest that these cases may represent a subtype of MS subjects.

Functional Systems Scores (FSS) are a good candidate for identifying clinical subtypes of MS. It is widely used in MS clinical trials and is divided into seven domains (pyramidal, cerebellar, brainstem, sensory, bowel and bladder, visual, and cerebral) (68). An asymmetric distribution of scores in these domains could identify subtypes of MS. Yang et al. (69) used a combination of a convolutional neural network and a rule-based natural language algorithm to accurately predict Kurtzke Functional System Scores (FSS) from the EHR notes of 4906 multiple sclerosis subjects. SUMMIT (Serially Unified Multicenter Multiple Sclerosis Investigation) is an international effort to create a repository of deeply phenotyped MS subjects utilizing standardized neurological examinations and the Kurtzke FSS (12). However, no subtypes based on FSS have been reported. Similarly, Dahlke et al. (70) examined the clinical course in 34,987 MS patients who had entered into clinical trials (31,863 with relapsing-remitting MS, 1873 with secondarily progressive MS, and 986 with primary progressive MS) but did not characterize MS subjects further as to clinical phenotype. Other ongoing longitudinal studies have been undertaken to characterize MS clinical phenotypes (16, 71) but they have not yet yielded new subtypes.

The increment in neurological deficits after MS relapses has been investigated (53–55, 57–59). Increasing disability in some subjects has been linked to the accumulation of pyramidal, sensory, cerebellar, and sphincter abnormalities (53–55). Furthermore, in some subjects relapses tend to occur at the same anatomical site as previous attack, and this is especially so for the optic nerve, spinal cord, brainstem, and cerebellum sites (57–59), suggesting that neurological signs and symptoms could accumulate at those affected areas. If relapses recur at sites of the previous attacks, this could foster subtypes of MS based on repeated relapses at the same anatomic site.

A network (also called a graph) is an assembly of nodes that are interconnected by edges (52). When all connected nodes come from the same class, the graph is unipartite. When each node is connected to a node of a second class, the graph is bipartite (72). Networks can be partitioned into communities of like nodes (also called clusters) (73, 74). Barabási (75) defines a community as “a locally dense connected subgraph in a network (page 325),” and that “… we expect nodes that belong to a community to have a higher probability of linking to other members of that community than to nodes that do not belong to the same community ….” Some of the partitioning algorithms depend upon the maximization of modularity which measures how well each community is separated from other communities.

Network analysis has proven useful in visualizing complex relationships between the phenotypes, genes, proteins, and metabolic pathways that underlie human diseases (76–81). Network analysis has provided important insights in into brain connectivity, and neuroimaging (82, 83). Network analysis has identified potential genetic causes of autism (84) and has clustered autism subjects by phenotype (85, 86). Network analysis has been used to identify genes that govern MS susceptibility (87–89), proteins implicated in the etiology of MS (90), as well as brain areas that undergo disconnection in MS (45, 91–93).

### Proposed approach

**Figure 1:**
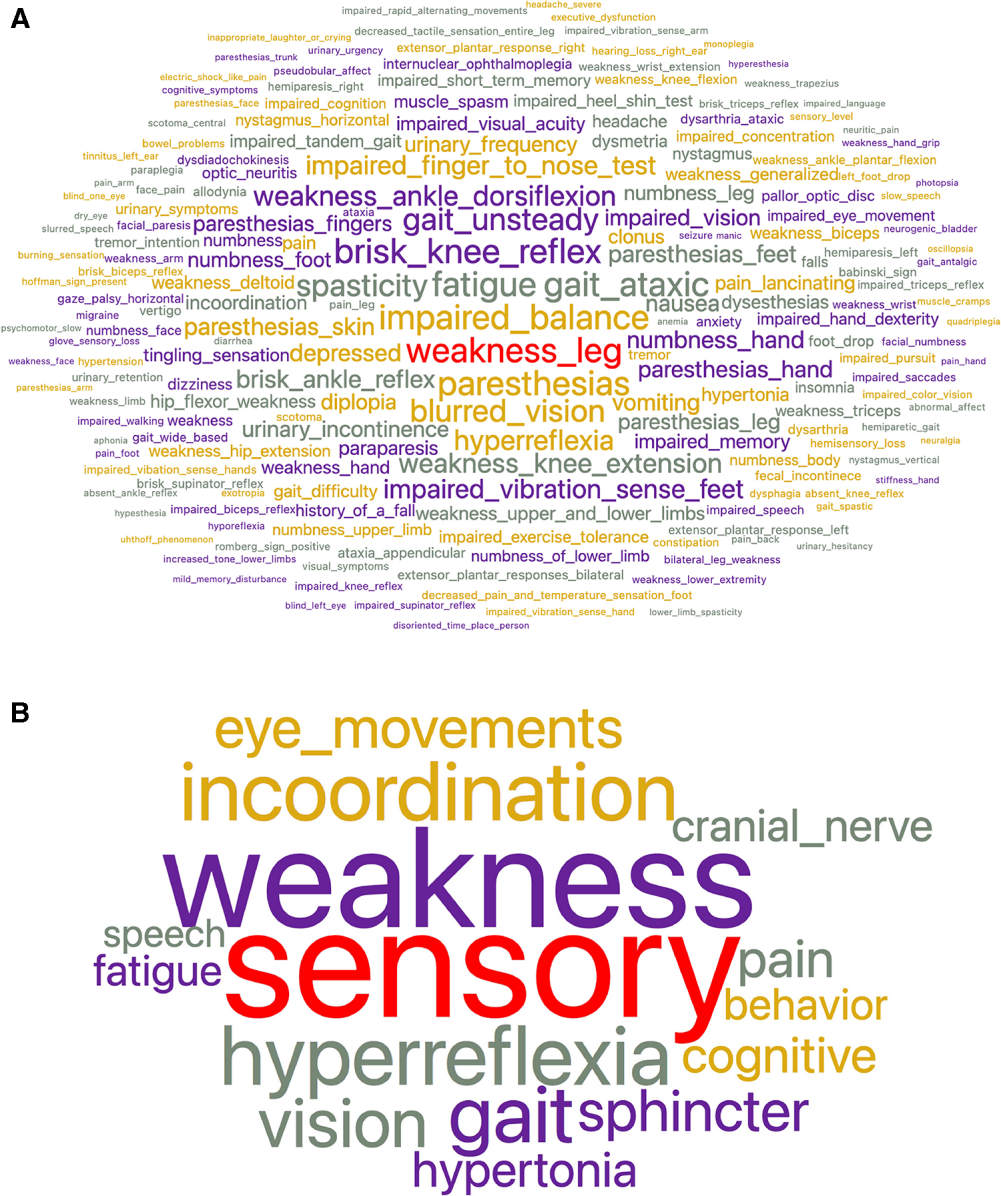
(**A**) Word cloud representing the frequency of signs and symptoms in the entire MS cohort before subsumption. Word size is proportional to frequency. There were 244 unique signs and symptoms. The most frequent signs and symptoms were leg weakness, impaired balance, fatigue, and paresthesias. Supporting files available on the project’s GitHub site. (**B**) Word cloud representing the frequency of signs and symptoms in the entire MS cohort after subsumption into 16 superclasses. Word size is proportional to frequency. The largest superclasses are sensory, weakness, hyperreflexia, and incoordination. Supporting files available on the project GitHub site.

The review of prior work suggested that there is a gap in identifying subtypes of MS based on signs and symptoms. Our goal was to identify clinical subtypes of RRMS using network analysis after feature reduction. We found 244 unique neurologic signs and symptoms in a cohort of 113 subjects with relapsing-remitting MS, mapped them to classes in a neuro-ontology, and then collapsed the classes into sixteen superclasses (Figures 1A,B). For each subject, the count of signs and symptoms in each superclass was normalized. A bipartite graph was created using NetworkX, with each subject node connected to one of sixteen superclass nodes by an edge proportional to the normalized count of signs and symptoms. Distances between subjects were calculated by the cosine similarity of their signs and symptoms. A unipartite graph was created in NetworkX where the nodes were subjects, and the edges were inter-subject distances. The unipartite and bipartite graphs were visualized in Gephi and partitioned into communities based on the Louvain algorithm (94). Modularity scores were used to evaluate the quality of the partitions. We used feature maps to characterize the communities. This approach could lead to classifying MS patients by clinical phenotype and supplement the phenotyping of MS subjects by disease course.

## Methods

### Subjects

One hundred and twenty MS subjects followed at the University of Illinois-Neuroscience Center were enrolled in the University of Illinois at Chicago (UIC) Neuroimmunology Biobank between August 2018 and March 2020 (mean age 42.7±12.8 years, 73% female, 27% male, 58% Black, 42% White). The Biobank is approved by the Institutional Review Board (IRB) of the University of Illinois College of Medicine. All subjects provided informed written consent at enrollment. Subjects were between 18-80 years old and had a diagnosis of RRMS based on the 2017 McDonald criteria (95). Subjects had been recruited for a study of blood biomarkers in MS where RRMS was an inclusion criterion and progressive MS was an exclusion criterion. Seven subjects with normal neurological examinations were excluded from the analysis leaving a final study sample of 113 subjects.

### Neuro-phenotyping

**Figure 2:**
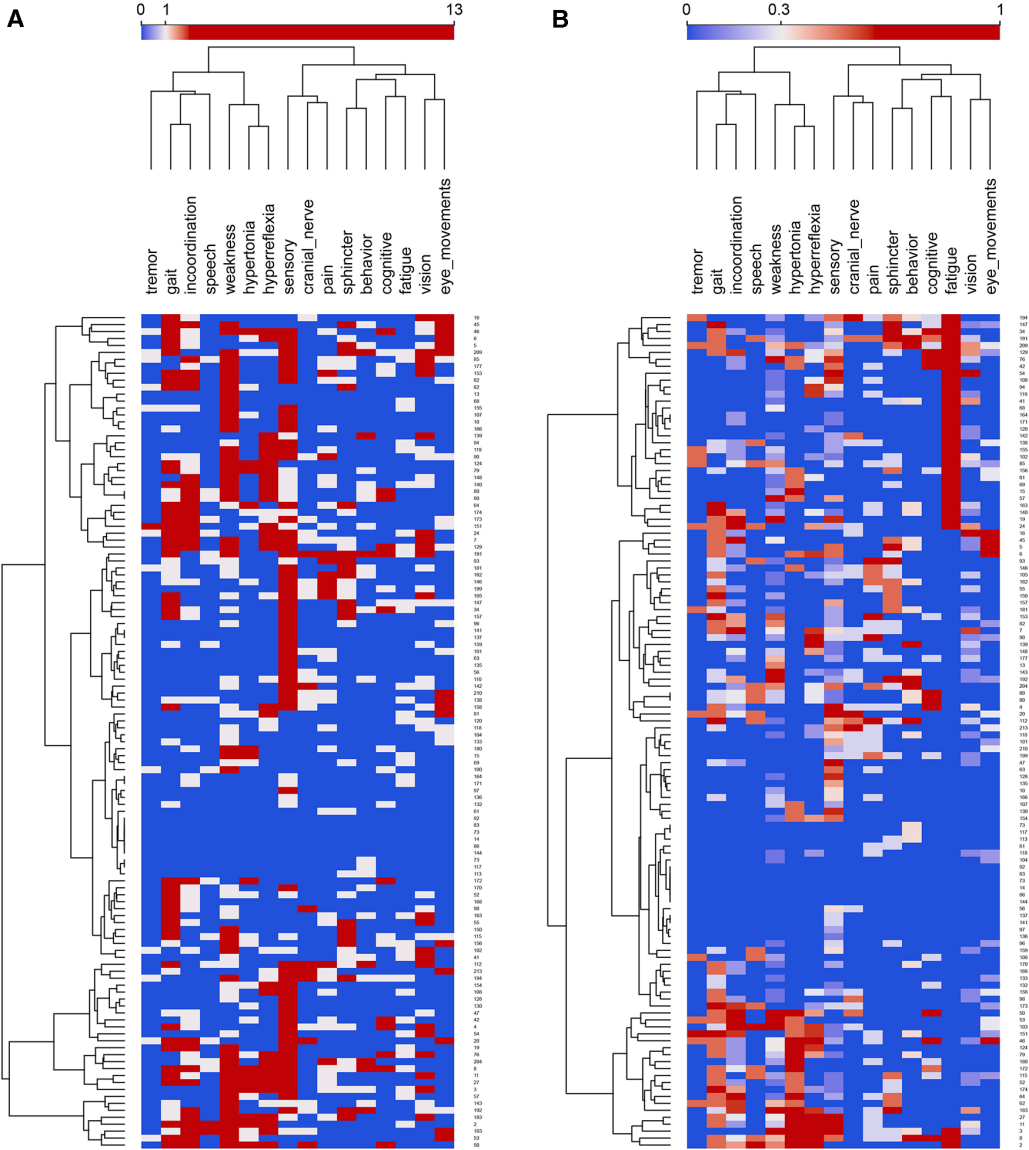
(**A**) Feature map of the entire cohort of MS patients before normalization. Rows are subjects, and columns are superclasses. Normalized feature counts in the columns range between 0 to 13 and the color scale is centered on 3 features. Rows and columns are clustered hierarchically with Ward linkage. Column distances by Pearson correlation coefficient; row distances are Euclidean. (**B**) Feature map of the entire cohort of MS patients after normalization. Rows are subjects, and columns are superclasses. Normalized feature counts in the columns range between 0 to 1 and the color scale is centered on 0.3 features. Rows and columns are clustered hierarchically with Ward linkage. Column distances by Pearson correlation coefficient; row distances are Euclidean.

The neurological progress notes from the electronic health record of all subjects were reviewed, and neurological signs and symptoms were recorded (11). The cumulative signs and symptoms (both active and resolved) of each subject were recorded and mapped to concepts in a neuro-ontology with 1,600 possible concepts (96). Subjects had 13.2∓9.2 signs and symptoms (mean ∓ standard deviation). The 113 subjects had 1,453 total signs and symptoms (244 unique signs and symptoms). Subsumption (97) was used to collapse the signs and symptoms (Figure 1A) into 16 superclasses (Figure 1B) that included *behavior*, *cognitive*, *cranial nerve*, *eye movement*, *fatigue*, *gait*, *hyperreflexia*, *hypertonia*, *incoordination*, *pain*, *sensory*, *speech*, *sphincter*, *tremor*, *vision*, and *weakness*. The largest superclasses were weakness, sensory, incoordination, and hyperreflexia. Each subject was represented as a 17-dimension vector where the first element of the vector was the case identification label, and the subsequent sixteen elements were the count for each of the sixteen superclasses (Figure 2A). Counts were normalized over the interval [0,1] using the *continuize* widget in Orange 3.32.0 (98) (Figure 2B). We chose to normalize counts because counts varied significantly between superclasses. For supporting data, see the project GitHub site.

### Network analysis, distance metrics, feature maps

Network analyses were performed on normalized 113×17 data arrays (89, 98, 99). NetworkX (100) converted the data arrays to GraphML files compatible with Gephi. Bipartite networks were visualized in Gephi 0.9.7 using a variety of layouts, with the final analysis using the Force Atlas layout with a repulsion force of 10,000. Visual inspection showed Force Atlas to have the optimal spacing of nodes and clarity of visualization. The bipartite network contained nodes of subjects and features (signs and symptoms) as nodes with a magnitude of the edges connecting subjects to features equal to the normalized feature score for each subject. In the bipartite networks, there were no direct subject-subject or feature-feature edges. Node sizes were proportional to the average weighted degree of each node. Communities were named based on their predominant features. Nodes were colored by their community membership, and colors were used consistently across graphs based on feature predominance. Edge widths were proportional to edge weight for the bipartite graphs. The unipartite networks were based on distances between subjects derived from the feature vectors for each subject. Distances were calculated in Orange using the *distances* widget for Pearson, Euclidean, and cosine distances. Visual inspection of the network graphs showed that the cosine-based graphs were superior to those based on the Pearson or Euclidean distances. Only the cosine distances were retained for further analysis (101). For the unipartite graphs, all nodes were subjects, and the edges were subject similarity based on the cosine distances. Node size was proportional to the degree (number of edges for each node). The edge width was fixed. Gephi was used to partition the unipartite and bipartite networks into communities based on the Louvain algorithm (94). The Louvain algorithm maximizes modularity (a measure of community separation). Modularity rises from 0.0 as the number of intra-community edges increases relative to inter-community edges. Larger values of modularity reflect a more robust separation of the communities. The degree, average degree, and modularity class for each node were calculated by Gephi. Modularity resolution was set to 1.0 for the unipartite graph and 1.15 for the bipartite graph. For the unipartite graph, two subjects were excluded from the final analysis as they formed communities with only one node. For the normalized unipartite graph, a cosine distance threshold of 0.4 was used to exclude weak edges. Feature means for each community were calculated by SPSS 28 (IBM, Chicago, IL). Differences between community feature means were tested by one-way ANOVA (SPSS). Feature maps were created with the *heat map* widget from Orange. The word cloud was created with the *word cloud* widget from Orange. The concordance for set membership between communities was measured by the Jaccard Index (102) where J is the Jaccard Index, and A and B are the set memberships of two communities:

$$J=\frac{A\cap B}{A\cup B}.$$

## Results

The largest superclasses of signs and symptoms in this cohort of MS subjects were sensory, weakness, incoordination, and hyperreflexia (Figure 1B). To prevent the superclasses of weakness and sensory from dominating the network analysis, the signs and symptoms were normalized on the interval [0,1] before network analysis and partitioning. Visual inspection of the feature map of the MS cohort suggested some clustering of subjects on signs and symptoms (Figures 2A,B) and that a network analysis to identify distinct communities would be fruitful.

**Figure 3:**
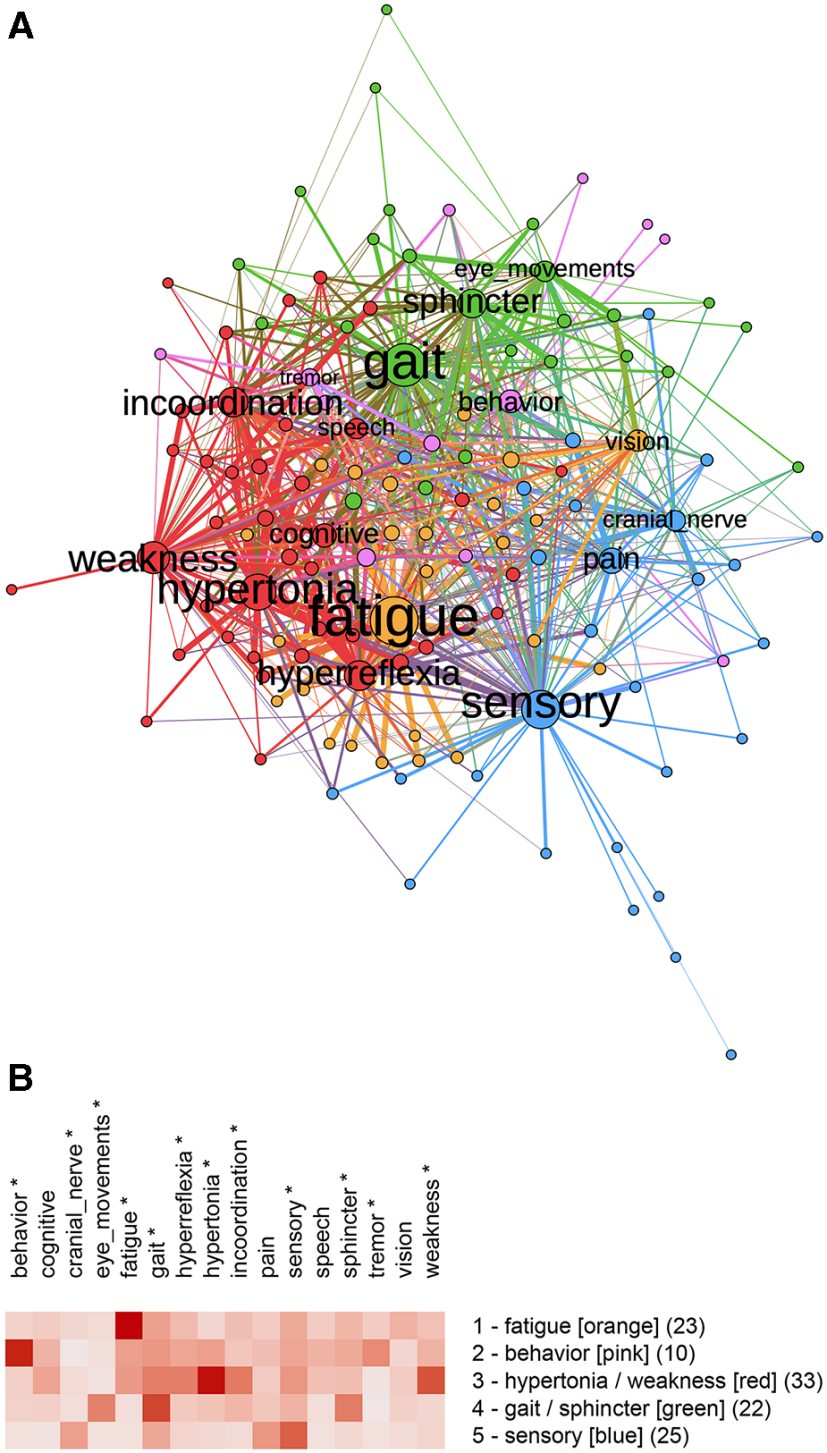
(**A**) Bipartite graph of normalized features. Labeled nodes are features; unlabeled nodes are subjects. Weighted connections (edges) are between features and subjects. (**B**) Feature map of the five communities identified by partitioning the bipartite graph with unnormalized features. Modularity analysis of the bipartite graph of normalized data showed five communities. Community 1 was predominantly fatigue, Community 2 was predominantly behavioral, Community 3 was weakness and hypertonia, Community 4 was gait and sphincter, and Community 5 was predominantly sensory features indicating features that differed significantly by the community (One-way ANOVA, p<0.05, df=4).

The bipartite graph was partitioned into five communities (Figure 3A) with a modularity score of 0.25. Communities were named and color-coded by the one or two features with the highest community means as **fatigue** (n=23), **behavior** (n=10), **hypertonia/weakness** (n=33), **gait/sphincter** (n=22), and **sensory** (n=25) (Figure 3B). ANOVA showed significant differences between communities for behavior (p<.001), cranial nerve (p=.008), eye movements (p<.001), fatigue (p<.001), gait (p=.029), hyperreflexia (p=.031), hypertonia (p<.001), incoordination (p=.002), sensory (p=.018), sphincter (p=.021), tremor (p=.006), and weakness (p=.001).

**Figure 4:**
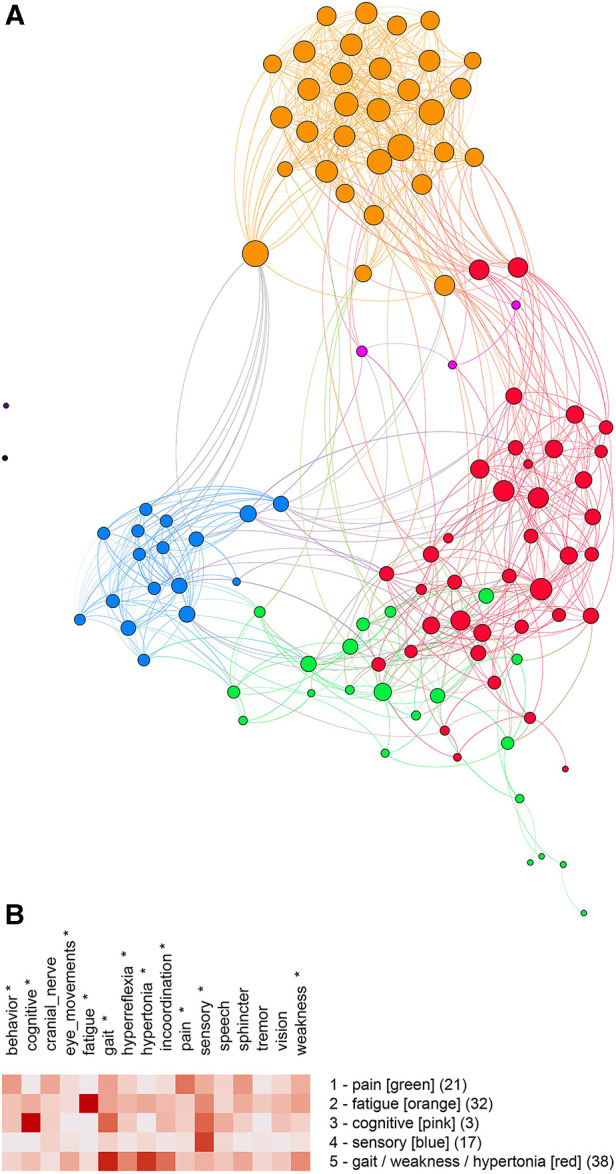
(**A**) Unipartite graph based on normalized features. Nodes are subjects, and node size is proportional to the number of edges. The largest communities are gait/weakness/hypertonia (red, n=38) and fatigue (orange, n=32). Note the small cognitive community (pink, n=3). (**B**) Feature map of the five communities identified by partitioning the unipartite network graph based on normalized features. Asterisks indicate features that differed significantly by the community (One-way ANOVA, p<0.05, df=4).

The unipartite graph was partitioned into five communities (Figure 4A) with a modularity score of 0.49. Communities were named by their predominant features: **pain**, **fatigue**, **cognitive**, **sensory**, and **weakness/gait/hypertonia** (Figure 4B). ANOVA analysis showed significant differences between communities for behavior (p=.033), cognitive (p<.001), eye movements (p=.047), fatigue (p<.001), gait (p<.001), hyperreflexia (p=.014), hypertonia (p<.001), incoordination (p<.001), pain (p<.001), sensory (p<.001), and weakness (p=.037).

Although partitioning the bipartite and unipartite graphs produced somewhat different communities, similarities between community membership and graphs were notable. We used the Jaccard Index (a set similarity measure) to assess the similarity between communities. Membership for the **fatigue** (J=0.72) and **sensory** (J=0.56) communities was similar for the unipartite and bipartite graphs. The unipartite graph community **gait/weakness/hypertonia** showed similarity to the bipartite graph communities **hypertonia/weakness** (J=0.36) and **gait/sphincter** (J=0.36). A complete table of Jaccard Index values is available on the project’s GitHub site.

## Discussion

Multiple sclerosis can present as sensory loss, weakness, incoordination, sphincter disturbance, diplopia, visual loss, cognitive impairment, fatigue, or even pain. We have used network analysis to identify distinct clinical subtypes of multiple sclerosis based on signs and symptoms. We first mapped the signs and symptoms of a cohort of multiple sclerosis subjects to concepts from neuro-ontology. We then created a bipartite graph, where subjects and their signs and symptoms were nodes in a graph (Figure 3A). In a bipartite graph, subjects are connected to signs and symptoms and not to other subjects. When the signs and symptoms of a subject are converted to vectors, distances between subjects can be calculated so that subject nodes can be connected to other subjects to form a unipartite graph (Figure 4A). Network analysis allowed us to identify communities of multiple sclerosis subjects who shared signs and symptoms in common. Partitioning of the unipartite and bipartite graphs based on modularity score identified communities with strong fatigue and sensory feature predominance. Both partitions had communities characterized by weakness combined with hypertonia or gait findings. Partitioning of the bipartite graph produced a small community with behavioral changes (depression, anxiety, etc.) and a gait/sphincter community. Partitioning of the unipartite graph produced a small community with cognitive findings and a medium-sized community with pain (Figure 4B).

Partitions of the unipartite graph yielded higher modularity scores than the bipartite graph, suggesting that the partitioning of the unipartite graph was more robust. The named communities for Figure 4B (**pain**, **fatigue**, **cognitive**, **sensory**, and **gait/weakness/hypertonia**) deserve special consideration as potentially identifiable multiple sclerosis subtypes. We found a strong overlap between the fatigue and sensory communities across both graphs as measured by the Jaccard Index. Significant overlap between the **gait/weakness/hypertonia** community from the unipartite graph with the **gait/sphincter** and **hypertonia/weakness** communities from the bipartite graph was noted. Although the partitioning of networks based on features suggests that identifiable MS subtypes may exist, variability across partitions does not permit a definitive characterization of subtypes.

Although we did not correlate community features with MRI findings, the communities detected may reflect the anatomic location of MS lesions (103, 104). Of particular interest is the tendency for relapses to occur at the sites of previous MS attacks (57–59). Recurring relapses at the same anatomic site could lead to increased symptoms in certain domains (e.g., weakness, incoordination, sphincter, visual, etc.) and make subtypes of MS more discernible. On the other hand, “pure” subtypes of MS (i.e., pure motor, pure sensory, pure cognitive) are uncommon; nearly all MS patients in our cohort have signs and symptoms in multiple symptomatic domains (see, for example, Figures 2A,B).

Two strengths of this study should be mentioned. First, community detection was done by network analysis which offers an alternative to unsupervised machine learning algorithms based on cluster analysis (105, 106). Second, we used subsumption and the hierarchical organization of signs and symptoms in an ontology to reduce the number of features used in the analysis (97). The current study demonstrates that subsumption can successfully group signs and symptoms of MS subjects into superclasses (Figures 1A,B). These superclasses can be used to characterize the clinical features of communities identified by network analysis.

The current study has several limitations. The sample size was small (N=113). The small sample size could cause a selection bias that influenced the communities found by network analysis. Network analysis of larger sample sizes may detect more robust communities with a different profile of predominant features. In particular, we did not identify communities of MS subjects with predominant vision, cranial nerve, or incoordination signs and symptoms, although such communities likely exist (15, 20–22). Another limitation was that we evaluated only one partitioning algorithm (Louvain). A limitation of the Louvain algorithm is that it does not exhaustively examine all possible partitions, so partitioning is non-deterministic, and partitions may change with each run (73, 107, 108). Other partitioning algorithms are available and might yield different results. We used subsumption to reduce the number of clinical features from 244 to sixteen. Different subsumption strategies would likely yield different results. We calculated distances between subjects using the cosine distance metric; other distance metrics are available and may have resulted in different results. Although the modularity scores of the partitions are comparable to those obtained on standard datasets like the Karate Club (73, 108), they are still modest (0.25–0.49). Another limitation was that subjects in the study were diagnosed with the RRMS phenotype. Without further analysis, our data cannot be extrapolated to other disease course phenotypes. Our analysis did not consider the race or sex of the subjects, which could influence clinical subtype (60, 109, 110). Finally, we partitioned MS subjects based on their accumulated signs and symptoms. Examining networks based on signs and symptoms at a single time would be instructive.

## Conclusions

MS phenotypes based on the clinical course are well-established. Clinical subtypes of MS based on clinical presentation are increasingly recognized. After mapping the signs and symptoms of a cohort of MS patients to classes in neuro-ontology and then collapsing these classes into sixteen superclasses, we used network analysis to identify clinical subtypes of MS based on signs and symptoms. Feature maps (Figures 3B, 4B) suggest that identifiable subtypes of MS with predominant signs and symptoms related to weakness, sensation, behavior, cognition, pain, and fatigue deserve further investigation. The clinical subtyping of MS subjects could supplement phenotyping by disease course. Additional studies may reveal that MS subtypes correlate with epigenetic, radiological, immunologic, or protein biomarkers.

## Data Availability

The raw data supporting the conclusions of this article will be made available by the authors, without undue reservation.
